# WHAT ARE THE PATHWAYS TO RETIREMENT: DO HEALTH AND COHORT MATTER?

**DOI:** 10.1093/geroni/igz038.2688

**Published:** 2019-11-08

**Authors:** Fang-Yi Huang, Min Li

**Affiliations:** 1 Trinity Washington University, College Park, Maryland, United States; 2 University of Florida, Gainesville, Florida, United States

## Abstract

Studies about retirement often neglect ethnic identity. Yet, racial and ethnic inequality plays an important role in retirement decisions. For instance, minorities are less likely to continue working in old age beyond the normal retirement age because of some individual factors such as poor or fair health, cognitive impairment, functional limitation (Green, 2005; Ghilarducci & Moore, 2015; Mudrazija, 2010), physical limitations (Radl, 2014; Solem et al., 2016; Pienta & Hayward, 2002), retirement benefit and eligibility of pension (Radl, 2014; Honig, 1996). This research examined the influence of ethnicity on whether working for people aged above 60 in Taiwan. In addition to testing the pathways to retirement, we scrutinized how occupational sector mediates the likelihood of ethnic effect on retirement. After comparing two cohorts from the dataset “Taiwan Longitudinal Study in Aging”, we found that working in public sector, white-collar workers, being a Mainlander, being non-married, higher education, self-reported bad health and functional limitation are all associated with the higher likelihood of retirement. Finding suggested that the incentive structure of the public pension for public sector workers, who are mostly Mainlanders, could contribute to the higher likelihood of retirement. However, the magnitude of mediator is much larger for the older cohort than the younger cohort. The study not only could benefit cumulative advantage approach by analyzing East Asia data but also bring policy implication, that retirement policy makers should reduce public pension incentive for public sector workers to delay the retirement age.

